# Prioritization of Epilepsy Associated Candidate Genes by Convergent Analysis

**DOI:** 10.1371/journal.pone.0017162

**Published:** 2011-02-24

**Authors:** Peilin Jia, Jeffrey M. Ewers, Zhongming Zhao

**Affiliations:** 1 Department of Biomedical Informatics, Vanderbilt University School of Medicine, Nashville, Tennessee, United States of America; 2 Department of Psychiatry, Vanderbilt University School of Medicine, Nashville, Tennessee, United States of America; 3 Bioinformatics Resource Center, Vanderbilt University School of Medicine, Nashville, Tennessee, United States of America; 4 School of Medicine, Vanderbilt University School of Medicine, Nashville, Tennessee, United States of America; 5 Department of Cancer Biology, Vanderbilt University, Nashville, Tennessee, United States of America; Leiden University Medical Center, Netherlands

## Abstract

**Background:**

Epilepsy is a severe neurological disorder affecting a large number of individuals, yet the underlying genetic risk factors for epilepsy remain unclear. Recent studies have revealed several recurrent copy number variations (CNVs) that are more likely to be associated with epilepsy. The responsible gene(s) within these regions have yet to be definitively linked to the disorder, and the implications of their interactions are not fully understood. Identification of these genes may contribute to a better pathological understanding of epilepsy, and serve to implicate novel therapeutic targets for further research.

**Methodology/Principal Findings:**

In this study, we examined genes within heterozygous deletion regions identified in a recent large-scale study, encompassing a diverse spectrum of epileptic syndromes. By integrating additional protein-protein interaction data, we constructed subnetworks for these CNV-region genes and also those previously studied for epilepsy. We observed 20 genes common to both networks, primarily concentrated within a small molecular network populated by GABA receptor, BDNF/MAPK signaling, and estrogen receptor genes. From among the hundreds of genes in the initial networks, these were designated by convergent evidence for their likely association with epilepsy. Importantly, the identified molecular network was found to contain complex interrelationships, providing further insight into epilepsy's underlying pathology. We further performed pathway enrichment and crosstalk analysis and revealed a functional map which indicates the significant enrichment of closely related neurological, immune, and kinase regulatory pathways.

**Conclusions/Significance:**

The convergent framework we proposed here provides a unique and powerful approach to screening and identifying promising disease genes out of typically hundreds to thousands of genes in disease-related CNV-regions. Our network and pathway analysis provides important implications for the underlying molecular mechanisms for epilepsy. The strategy can be applied for the study of other complex diseases.

## Introduction

Epilepsy is a brain disorder involving recurrent seizures of any type, and is one of the most common neurological disorders affecting young people. Despite numerous efforts to elucidate its pathological basis, the genetic and environmental factors underlying epilepsy have remained unclear, a fact that may be partially attributable to the complex subtypes of the disorder. Current studies in the field, including genome-wide association (GWA) studies of other neurological disorders, have suggested that rare variants, such as large deletions or duplications, may play important roles in the pathogenesis of similarly complex diseases [1].

A number of studies aiming to identify such rare variants have been conducted, and have successfully revealed several loci and genes conferring susceptibility to epilepsy, such as deletions at 15q13.3 [2] and 16p13.11 [3]. The association of copy number variations (CNVs) with epilepsy has gained recent attention, and has served to expand our knowledge of epileptic pathogenesis. However, these CNV regions are typically large and contain many genes, so it is not surprising that it still remains unclear which are responsible for the disease [4]. To effectively approach the prioritization of these candidate genes, one can incorporate evidence from both the transcriptome and proteome level, in order to investigate the genetic signals interactively and systematically [5]–[6].

To this end we performed an integrative analysis of CNV data, gene association data accessed via the HuGE Navigator [7], protein-protein interaction (PPI) data, and gene expression data. By applying a graphic algorithm, we constructed subnetworks for CNV genes and for previously studied genes separately, in the context of the whole human PPI network. A comparison of the genes comprising the two networks revealed 20 overlapping genes, which hold high priority as candidates for epilepsy. Most of these candidate genes could connect in a molecular network featuring gamma-aminobutyric acid (GABA) signaling and BDNF/MAPK signaling pathways. Incorporation of an expression dataset from Gene Expression Omnibus (GEO) further highlighted two genes, *CHRNA7* and *GABRA1*, which are differently expressed between epilepsy patients and controls. Finally, we performed pathway enrichment analysis of the two subnetworks, followed by examination of crosstalk between enriched pathways. These results showed that neurotransmitter related pathways, immune related pathways, and kinase regulatory pathways are significantly enriched and clustered into functional groups.

## Materials and Methods

### Data sources

Copy number variation data was collected from a recently published study, which conducted genome-wide screening of CNVs for epilepsy disorders [3]. A total of 3812 patients with a diverse spectrum of epilepsy syndromes and 1299 neurologically normal controls were genotyped primarily using the Illumina Human 610-Quad genome-wide genotyping array. We collected 373 genes (denoted as “CNV-genes”) that were located in the heterozygous deletion regions greater than 1 Mb observed in epilepsy patients (see Table 1 in ref. [3]). As reported in the original work, genes fully or partially covered by these regions were listed and thus used in our analysis.

**Table 1:** Function enrichment of the subnetwork genes.

Diseases and disorders	*P*-value^a^	#molecules
*CNV-subnetwork*		
Neurological disease	9.41×10^−11^–3.56×10^−5^	132
Cancer	2.22×10^−10^–5.70×10^−5^	102
Genetic disorder	1.23×10^−9^–7.60×10^−6^	176
Psychological disorders	1.23×10^−9^–3.24×10^−5^	57
Reproductive system disease	2.22×10^−9^–4.78×10^−5^	49
*HuGE-subnetwork*		
Neurological disease	2.02×10^−52^–5.62×10^−7^	143
Genetic disorder	4.47×10^−37^–5.15×10^−7^	165
Psychological disorders	4.34×10^−27^–4.79×10^−7^	81
Skeletal and muscular disorders	1.54×10^−25^–5.15×10^−7^	112
Organismal injury and abnormalities	9.49×10^−20^–5.92×10^−7^	54

a *P*-values were calculated by Fisher's exact test, indicating probability of the association of the candidate genes with the canonical pathway from chance. Each disease category has several pathways, thus, the range of their *P*-values is provided.

The HuGE Navigator is an integrated knowledge base of human genome epidemiology, focusing primarily on the continual collection of epidemiologic and genetic studies, including publications of genetic variants, gene-disease associations and gene-gene or gene-environment interactions [7]. We specifically searched the Phenopedia page for epilepsy related association studies and reported genes. Using the exact match of term “epilepsy”, we retrieved 165 genes annotated in the HuGE database (as of April 23, 2010). We also checked other epilepsy related keywords (e.g., “epilepsies, myoclonic”) to search the HuGE database and could only find a small number of genes, most of which were already included in the 165 gene list. Thus, the 165 genes (hereafter denoted as “HuGE-genes”) were included in our analysis. Specifically, Heinzen's data was not included in the HuGE database at the time we retrieved the data, thus, the two gene sets are independent. Of note, the HuGE database collects published genetic studies without distinguishing positive or negative associations. Because replication rate of association is very low in psychiatric genetics [8], and we attempted to include potential candidate genes for convergent analysis, we included all of them in this work.

We downloaded a comprehensive human PPI dataset from the PINA platform (http://csbi.ltdk.helsinki.fi/pina/). PINA collected and manually curated PPI data from six major PPI databases (HPRD, IntAct, DIP, MINT, BioGRID, and MIPS/MPact) and its data was derived from high-throughput experiments and literature supported by experiments. Approximately 10,000 nodes and ∼50,000 interactions were included in the dataset as of August, 2009. We used this dataset to construct a human PPI network (i.e., interactome), upon which we performed follow-up subnetwork analysis.

### Subnetwork construction

We sought to identify connections among the CNV-genes and HuGE-genes, hypothesizing that genes susceptible to epilepsy would not be working independently, but instead would interact with one another. We used the Steiner minimum tree algorithm [9] to search for subnetworks in the human PPI network to highlight genes of interest. The Steiner tree algorithm was initially designed for searching a weighted network, and aims to determine the least cost connected subgraph spanning a gene subset of special interest. A detailed description of the algorithm can be found in the original work [9]. In our application, since our PPI network is unweighted, the execution of Steiner tree was simplified to construct a connected subgraph spanning a maximum proportion of our interesting nodes, e.g., the encoded proteins of CNV-genes or HuGE-genes, and impeded by the restriction of minimizing the nodes not belonging to the interesting nodes.

### Integration with expression data

To integrate gene expression information, we downloaded an expression dataset from GEO (http://www.ncbi.nlm.nih.gov/geo/, accession number: GSE20977). This dataset was also part of Heinzen's work [3]. Blood samples from 7 epilepsy patients with 16p13.11 heterozygous deletions and 8 normal controls were studied to estimate gene expression levels using Illumina Human HT-12 v3 microarrays [3]. The initial aim of this dataset was to evaluate the impact of the deletion on gene expression. The data had been previously normalized using robust spline normalization, and probes with the maximum intensity were chosen to represent each gene. We used a two-sided *t*-test to identify significant differentially expressed genes between epilepsy patients and controls.

### Pathway enrichment and pathway crosstalk

We used the Ingenuity Pathway Analysis (IPA) system (http://www.ingenuity.com) to perform pathway enrichment tests. To compute a *P* value for each pathway, IPA implements Fisher's exact test. We defined significant pathways as those with the number of genes of interest ≥5 and *P* value <0.01.

For pathway crosstalk, we considered paired pathways with no less than 3 overlapping genes. To measure the gene-set overlap score, we followed the algorithm proposed by enrichment map, a package for Cytoscape [10], and investigated two measures of gene-set overlap: the Jaccard Coefficient 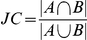, and Overlap Coefficient 
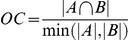
, where *A* and *B* denote two pathways. We took the average of the two measurements as the score defining pathway crosstalk, represented visually as a graph [11].

## Results

**Figure 1:**
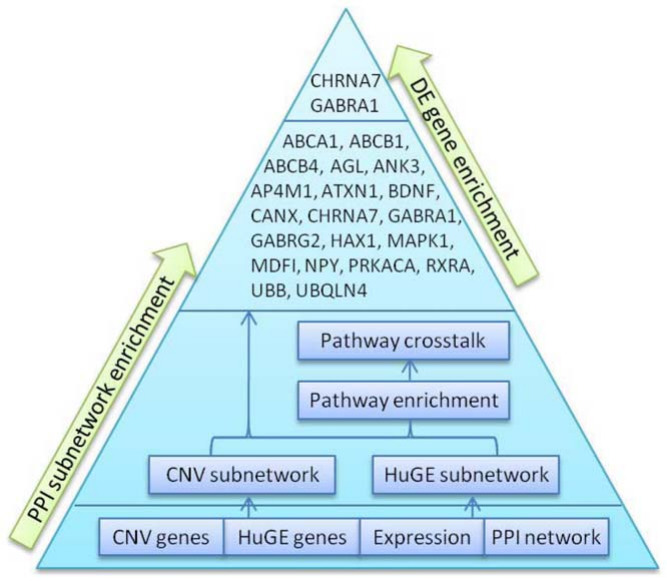
Convergent functional analysis of CNV genes for epilepsy. PPI: protein-protein interaction. DE gene: differently expressed genes in epilepsy patients and normal individuals.

Figure 1 illustrates our stepwise procedure to reduce the pool of CNV-genes. Starting with two gene sets (i.e., CNV-genes and HuGE-genes), we first constructed subnetworks for either gene set by incorporating an established human interactome and implementing a Steiner minimum tree algorithm. Comparison of the two subnetworks revealed 20 overlapping genes of high priority. We investigated the function and relationships of these 20 genes, and further incorporated gene expression data to highlight two genes deemed by our evidence as most relevant to epilepsy. To further provide insights on the functional level, we then performed pathway enrichment of genes in the resultant subnetworks, examined crosstalk quantitatively between significant pathways, and organized them in a graphical functional map.

### Subnetwork construction of CNV-genes and HuGE-genes

There are 373 CNV genes reported in Heinzen's work [3]. The constructed CNV-subnetwork using the Steiner minimum tree algorithm contains two types of nodes: 149 are of CNV-genes that could be connected using the Steiner tree algorithm, and 126 were additionally recruited in the process of subnetwork construction. The remaining CNV-genes either lacked annotated interactions with other proteins in the PPI network or would have included too many unrelated nodes to connect them, thus, they were not included for further analysis.

The same procedure was performed for HuGE-genes. As of April 23, 2010, there were 165 genes annotated by HuGE as related to epilepsy. Using the Steiner tree algorithm, we analyzed these genes and constructed a HuGE-subnetwork containing 202 nodes, 127 of which belonged to the initial HuGE-gene list, while 75 were recruited additionally.

### High-priority candidate genes from both subnetworks

On the gene level, we identified 20 genes in common between the two subnetworks, and assigned them high-priority (Table 1) as candidate genes for epilepsy. Five genes, *ABCB1*, *ABCB4*, *CHRNA7*, *GABRA1*, and *GABRG2*, are located within reported CNV regions and have been studied previously for their association with epilepsy as collected by HuGE. *AGL* and *AP4M1* were found only within CNV genes, while *ATXN1*, *BDNF* and *NPY* were annotated solely in HuGE. The other genes are neither located in CNV regions nor collected by HuGE. They were identified during the process of constructing the subnetworks; thus, they are important for the structure of both subnetworks. Of note, the gene ankyrin 3, node of Ranvier (ankyrin G), or *ANK3*, has been reported in previous GWA studies to be associated with schizophrenia [12] and bipolar disorder [13].

**Figure 2:**
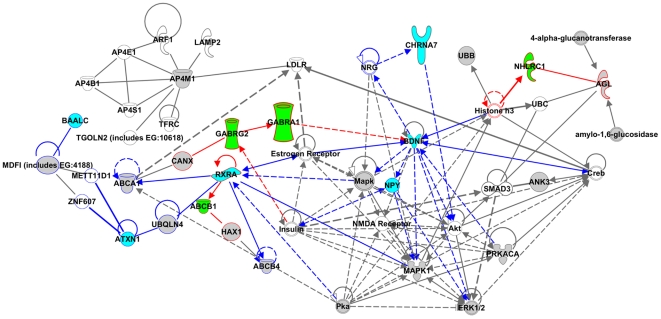
Molecular network of high-priority genes. Shaded nodes are genes from both CNV and HuGE networks. Green nodes are annotated in association with epilepsy. Cyan nodes are annotated in association with schizophrenia. Red edges are from epilepsy nodes to nearest-neighbors. Blue edges are from schizophrenia nodes to nearest-neighbors.

Using the Network Analysis tool in the IPA system, we observed that 17 of the overlapping 20 genes appear in the top network, which is significant for functional association with “carbohydrate metabolism, lipid metabolism, and molecular transport” (Figure 2). Three genes, *UBB*, *GABRA1*, and *GABRG2*, are constituents of the GABA signaling pathway and are connected with other molecules through protein-protein interaction (e.g., GABRG2 and CANX) or regulation of gene expression (e.g., GABRA1 was observed to decrease the expression of BDNF). We also observed molecules and complexes such as BDNF, MAPK1, PRKACA, and the NMDA receptor, which are known to participate in diverse neurodevelopmental processes. Interestingly, several hormone-related proteins and hormone receptors were also included in the network, including the estrogen receptor and neuropeptide Y (NPY).

### CHRNA7 and GABRA1 as differently expressed genes

We used the microarray data to compare gene expression levels between epilepsy patients and normal individuals. A two-sided *t*-test was performed to identify genes showing significant differential expression (DE genes). We identified 1278 genes having *P* < 0.05, including three genes discussed in the original study: *NDE1*, *C16orf63* and *KIAA0430* [3].

To further prioritize candidate genes for epilepsy, we examined the expression levels of the genes in the subnetworks. A few of these genes were found to be significantly differentially expressed between epilepsy patients and normal controls, including 12 genes from the CNV-enriched subnetwork and 12 genes from the HuGE subnetwork. Interestingly, two of these genes appear in our list of high-priority genes, namely *CHRNA7* (*P* = 0.014) and *GABRA1* (*P* = 0.016).

### Pathway enrichment and crosstalk

We used the Ingenuity Pathway Analysis (IPA) system to analyze the CNV-subnetwork and HuGE-subnetwork, respectively. In the “Disease and Disorder” category of IPA system, “neurological disease” is the most significant category for both CNV-subnetwork and HuGE-subnetwork (Table 2), supporting the hypothesis of the involvement of neurological dysfunction in epilepsy. We also observed “genetic disorder” and “psychological disorders” as significant in both networks. While these results are expected for the HuGE-genes, which were derived from previous studies that often tested the neurological hypothesis of this disease, the findings based on CNV-genes, which derived from an unbiased genome-wide CNV dataset published recently and have not been included in IPA yet, are important for understanding the molecular mechanism of epilepsy.

**Table 2:** Evidence of high-priority candidate genes for epilepsy.

Gene symbol	Gene name	CNV-gene	HuGE-gene
*ABCA1*	ATP-binding cassette, sub-family A (ABC1), member 1		
*ABCB1*	ATP-binding cassette, sub-family B (MDR/TAP), member 1	Y	Y
*ABCB4*	ATP-binding cassette, sub-family B (MDR/TAP), member 4	Y	Y
*AGL*	Amylo-alpha-1, 6-glucosidase, 4-alpha-glucanotransferase	Y	
*ANK3*	Ankyrin 3, node of Ranvier (ankyrin G)		
*AP4M1*	Adaptor-related protein complex 4, mu 1 subunit	Y	
*ATXN1*	Ataxin 1		Y
*BDNF*	Brain-derived neurotrophic factor		Y
*CANX*	Calnexin		
*CHRNA7*	Cholinergic receptor, nicotinic, alpha 7	Y	Y
*GABRA1*	Gamma-aminobutyric acid (GABA) A receptor, alpha 1	Y	Y
*GABRG2*	Gamma-aminobutyric acid (GABA) A receptor, gamma 2	Y	Y
*HAX1*	HCLS1 associated protein X-1		
*MAPK1*	Mitogen-activated protein kinase 1		
*MDFI*	MyoD family inhibitor		
*NPY*	Neuropeptide Y		Y
*PRKACA*	Protein kinase, cAMP-dependent, catalytic, alpha		
*RXRA*	Retinoid X receptor, alpha		
*UBB*	Ubiquitin B		
*UBQLN4*	Ubiquilin 4		

**Figure 3:**
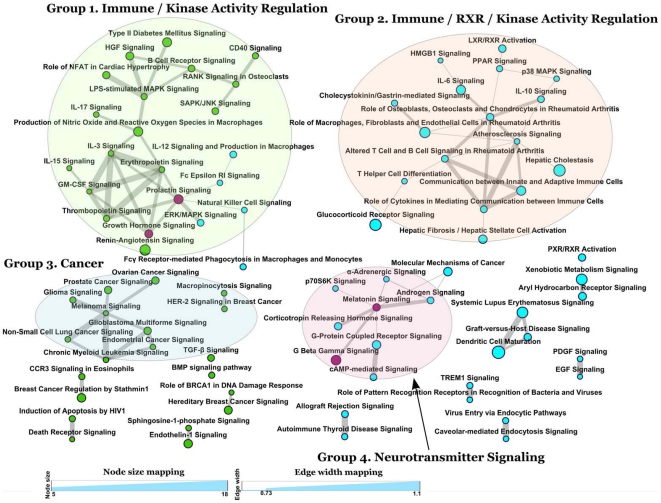
Pathway crosstalk and functional map of the CNV-subnetwork and HuGE-subnetwork. We indicate each pathway as a gene set. Significant pathways (nodes) are connected based on their overlap of component genes and are represented as edges. Node color indicates gene set membership, i.e., green pathways are enriched in the CNV-subnetwork; blue pathways are enriched in the HuGE-subnetwork; purple pathways are enriched in both subnetworks. Node size is proportional to the total number of encoded proteins of CNV-genes and HuGE-genes mapped in each pathway. Edge width is proportional to the overlap score of the related pathways (see Materials and Methods). The legend in the bottom shows the node size (i.e., the number of interesting proteins in each pathway) and edge width (i.e., the extent of the overlap between two nodes).

In the pathway enrichment analysis, 129 canonical pathways were nominally enriched in the CNV-subnetwork (Fisher's exact test, *P* < 0.01 and number of interesting genes ≥5, Table S1), and 75 in the HuGE-subnetwork (Table S2). We further investigated the relationships among these pathways to identify general biological features of epilepsy genes. Specifically, we examined crosstalk among these pathways by computing overlap scores based upon shared genes between any two pathways, and represented them graphically using Cytoscape. In Figure 3, nodes represent significant pathways and edges indicate crosstalk between them. A connection (i.e., represented as an edge) is established if two pathways share ≥3 genes, and is assigned a weight as the corresponding overlap score (see Materials and Methods), represented by the width of the edge in Figure 3. To highlight highly related pathways, only the top scored connections and corresponding pathways are shown in Figure 3. For CNV pathways, top scored connections are those scored within the top 1%, while for HuGE pathways we included the top 5% because the number of significant pathways is less than that of the CNV-subnetwork. Figure 3 includes the resulting 40 pathways and 44 connections/edges from CNV-pathways and 47 pathways and 48 connections from HuGE-pathways. Four of the pathways were overlapped.

Four groups were automatically formed by our analysis of pathway crosstalk, which we termed “functional groups”. As shown in Figure 3, group 1 and group 2 are formed by the CNV- and HuGE-subnetworks respectively, and each contains several immune pathways and kinase regulatory pathways. IL-3 signaling, IL-15 signaling, and IL-17 signaling were significantly enriched in the CNV-subnetwork and clustered in group 1. Their crosstalk involved JAK2, MAPK1, PIK3R1, and PIK3R3, which appear in all three pathways and are included in the CNV-subnetwork. Similarly, in group 2, which mainly consisted of HuGE-pathways, IL-6 and IL-10 signaling pathways were observed and found to be connected through the pathway “role of macrophages, fibroblasts and endothelial cells in Rheumatoid Arthritis”. No edge is indicated between the IL-6 and IL-10 pathways because their overlap score failed to meet the cutoff, but they share a number of common genes, including *IL1A*, *IL1B*, *IL1R1*, *IL1RN*, *IL6*, and *TNF*. Group 1 also contains the ERK/MAPK signaling pathway, a pathway known to work in tandem with the BDNF/Trk signaling pathway to regulate gene expression and trafficking during central nervous system (CNS) development [14]–[15]. Within group 2, the p38 MAPK signaling pathway was also observed. The HuGE-subnetwork also formed a group including neurotransmitter pathways, such as the G-protein coupled receptor and cAMP-mediated signaling pathways, and androgen signaling pathways (group 4). The CNV-subnetwork formed a group of several cancer pathways (group 3), which might be due to the tendency of cancer genes to have more interactions with other proteins in the PPI network, thus influencing their recruitment during CNV-subnetwork construction.

## Discussion

We collected four types of high-throughput datasets for epilepsy disorders and performed integrative analysis using bioinformatics approaches. Following a stepwise enrichment procedure, we constructed PPI subnetworks enriched with CNV genes or previously studied candidate genes for epilepsy. The overlap between the two subnetworks comprised 20 genes, 17 of which were included in a molecular network relevant to GABA signaling and neurodevelopmental events. Interestingly, two of the overlap genes, *CHRNA7* and *GABRA1*, were also shown to be significantly differentially expressed between epilepsy patients and normal controls. Furthermore, functional enrichment of the two subnetworks and examination of pathway crosstalk highlighted shared “neurological disease” in the disease category and immune/kinase-regulatory/neurotransmitter related pathways in the canonical pathway category.

In the molecular network containing 17 of the 20 high priority genes (Figure 2), we observed GABA signaling related genes, BDNF/MAPK pathway genes, estrogen receptor and NPY and their intimate interactions. Three genes from the GABA signaling pathway, including two GABA A (GABA_A_) receptor subunits, *GABRA1* and *GABRG2*, were included. GABA is well studied as the primary inhibitory neurotransmitter in mammalian brain, and synaptic inhibition is largely mediated by fast activation of GABA_A_ receptors. Seizure is associated with imbalance of excitatory and inhibitory synaptic activities, the latter of which is believed to be associated with GABA, and deregulated expression and altered function of GABA_A_ receptors has been previously observed in different types of epilepsy. For example, increased expression of GABA_A_ receptors was observed in the hippocampus of patients with temporal lobe epilepsy [16]–[17], and an Ala322Asp mutation in GABRA1 was found in juvenile myoclonic epilepsy patients [18]–[19]. Additionally, many current pharmacological treatments for epilepsy target elements of the GABA signaling pathway, including GABA receptors and transporters. The observation of *GABRA1* and *GABRG2* in our network provides strong evidence of the association between GABA_A_ receptors and epilepsy. *GABRA1* was also identified to be differently expressed between epilepsy patients and controls in our analysis of expression data.

BDNF is one of the neurotrophins, a large family that promote the growth, survival, and differentiation of cells in the CNS. These actions of neurotrophins are mediated by several cell surface receptors including TrkA, TrkB and TrkC, which in turn activate G-proteins and signaling cascades through intracellular tyrosine kinase receptors and ultimately induce downstream gene expression and regulation. In our subnetwork, we observed an active regulatory relationship between BDNF and MAPK1, which is also a member of Erk1/2 dimer in MAPK/ERK signaling pathways. These on some level reflect the complex role that BDNF may play in epilepsy, e.g., increased expression of *BDNF* during seizures of temporal lobe epilepsy patients as well as animal models have been observed frequently, although it is not clear whether seizures or injury increase expression of *BDNF*, or whether BDNF promotes epileptogenesis by increasing excitability [20].

Within our top-scoring network (Figure 2), we also observed the estrogen receptor and NPY. These inclusions are interesting given prior hypotheses regarding the role of estrogen and neuropeptide signaling in seizure disorders [21]–[22]. Estrogen, in particular, has been previously implicated as a modulator of GABAergic function [23]. Indeed, many antiepileptic therapies are known to interact with estrogen signaling by inhibiting aromatase (e.g., oxacarbazepine, phenobarbital, phenytoin, valproate) [24], linking estrogens to globulins (e.g., carbamazepine) [25], or directly binding to estrogen receptors (e.g., gabapentin) [26]. NPY, also included in the network, has been shown to co-localize and interact with estrogen [27]–[28]. NPY has been shown to play an important role in GABAergic interneuron regulation of excitability, and has been demonstrated to have neuroprotective effects against kainate-induced excitotoxicity, both *in vivo* and *in vitro* [29]–[30]. Further investigation of estrogen and *NPY* in relation to epilepsy may be promising in the search for effective antiepileptic therapies.

Interestingly, within the network, we also observed several genes previously reported to be associated with schizophrenia, including *ABCB1*, *ANK3*, *ATXN1*, *BDNF*, *CHRNA7*, *GABRA1*, *GABRG2*, *NPY*, and *PRKACA* [31], indicating their potential co-morbidity. The co-morbidity between schizophrenia and epilepsy has been aware for a long time by many aspects of the two diseases. For example, genetic studies have shown that the human leucine-rich, glioma inactivated family genes were linked to both epilepsy and schizophrenia [32]; and anticonvulsant drugs have been widely used in the treatment of psychiatric disorders. Among the genes we identified, mutation of *CHRNA7* has been observed in previous schizophrenia studies [33]. *CHRNA7* locates in 15q13.3, a region having recurrent deletion reported in epilepsy [2], late-onset Alzheimer's disease [34], mental retardation [35], seizures [35], and autism [36], among other pathologies. Furthermore, *ANK3* has been found to be associated with schizophrenia [12] and bipolar disorder [13]; however, to our knowledge, it has not been well studied in epilepsy yet. These results may further help us to understand the relations between epilepsy, schizophrenia, and other psychiatric disorders.

Our pathway enrichment analysis of the two subnetworks and the follow up investigation of pathway crosstalk revealed several functional groups of interest. Of note, the pathway crosstalk map showed more similarity between the two subnetworks on the level of pathways and functional mechanisms than observed at the gene level. For example, groups 1 and 2 consist of pathways from different subnetworks, yet share a number of immune-related pathways. These observations indicate the common involvement of immune, neurotransmitter, and cell signaling pathways in epilepsy, and reveal that close interactions between these pathways allow for a system of complex regulation.

While genes from our CNV analysis were identified from large-scale genotyping of epilepsy patients and can be considered as *de novo*, the HuGE genes have been studied in previous publications and are thus “known” candidate genes. The subset of genes common to both lists is of particular interest, as it represents a consensus between multiple lines of evidence. However, the original gene lists shared only 5 genes, and their interrelationships remain unclear. Using our network based method, we expanded this overlap to 20 genes in the context of a PPI network, and further demonstrated their relationship in a small molecular network.

Many methods and approaches have been proposed to identify disease candidate genes, such as single variant or gene analysis, haplotype analysis, meta-analysis of variants, epistatic analysis, genome-wide associate studies (GWAS), multiple dimensional evidence-based approaches, and network and pathway approaches [37]–[39]. While each of these methods has its advantages (e.g., GWA studies can examine one to several million SNPs at one time), it also has some disadvantages. For example, analysis of individual variants or genes could only detect genetic signal at one specific locus. Many genes are expected to be involved in complex diseases such as psychiatric disorders, but their risk might be too moderate or weak to be detected in a typical association study. On the other hand, most computational approaches to identifying or predicting disease genes require a pre-defined set of “golden standard genes”[40]. Comparing with those methods, our convergent strategy does not rely on golden standard genes, while it examines many genes simultaneously. Although it has limitations such as incompleteness of the dataset, it provides an alternative and potentially effective approach to identifying disease candidate genes from a variety of genetic and genomic datasets.

As above, a potential limitation of our work is the incompleteness of the datasets we used. For example, caution should be taken when using all the available “disease genes” in the HuGE database, as some might represent negative results and cause noise in convergent analysis. Furthermore, the CNV regions used in our study were greater than 1 Mb in length. There might also be important genes in smaller regions or in deleted regions, which were not immediately available from the original publication [3]. However, the CNV regions we used in the current study were directly from the original publication and were of high quality [3]. Nevertheless, our convergent strategy was able to identify 20 high-priority genes within strong biological context. Inclusion of more complete datasets or other types of datasets in future will greatly improve the quality of the current work.

In conclusion, we proposed a stepwise enrichment procedure to converge CNV genes by incorporating publically available high-throughput datasets. By applying a subnetwork construction algorithm, we established a subnetwork for CNV genes, as well as for a set of previously reported genes for epilepsy. The overlap between the two subnetworks constitutes a high-priority candidate gene set for epilepsy. Integration of additional gene expression data further narrowed the candidate gene list to two of special interest. This procedure is extensible to other disorders.

## Supporting Information

Table S1 Significant pathways in CNV-subnetwork.
(DOC)

Table S2 Significant pathways in HuGE-subnetwork.
(DOC)

## References

[1] Cirulli ET, Goldstein DB (2010). Uncovering the roles of rare variants in common disease through whole-genome sequencing.. Nat Rev Genet.

[2] Helbig I, Mefford HC, Sharp AJ, Guipponi M, Fichera M (2009). 15q13.3 microdeletions increase risk of idiopathic generalized epilepsy.. Nat Genet.

[3] Heinzen EL, Radtke RA, Urban TJ, Cavalleri GL, Depondt C (2010). Rare deletions at 16p13.11 predispose to a diverse spectrum of sporadic epilepsy syndromes.. Am J Hum Genet.

[4] Mefford HC, Eichler EE (2009). Duplication hotspots, rare genomic disorders, and common disease.. Curr Opin Genet Dev.

[5] Kurian SM, Le-Niculescu H, Patel SD, Bertram D, Davis J (2009). Identification of blood biomarkers for psychosis using convergent functional genomics..

[6] Sun J, Jia P, Fanous AH, van den Oord E, Chen X (2010). Schizophrenia gene networks and pathways and their applications for novel candidate gene selection.. PLoS ONE.

[7] Yu W, Gwinn M, Clyne M, Yesupriya A, Khoury MJ (2008). A navigator for human genome epidemiology.. Nat Genet.

[8] Sun J, Kuo PH, Riley BP, Kendler KS, Zhao Z (2008). Candidate genes for schizophrenia: a survey of association studies and gene ranking.. Am J Med Genet B Neuropsychiatr Genet.

[9] Klein P, Ravi R (1995). A nearly best-possible approximation algorithm for node-weighted Steiner trees.. J Algorithms.

[10] Cline MS, Smoot M, Cerami E, Kuchinsky A, Landys N (2007). Integration of biological networks and gene expression data using Cytoscape.. Nat Protoc.

[11] Pinto D, Pagnamenta AT, Klei L, Anney R, Merico D (2010). Functional impact of global rare copy number variation in autism spectrum disorders.. Nature.

[12] Athanasiu L, Mattingsdal M, Kahler AK, Brown A, Gustafsson O (2010). Gene variants associated with schizophrenia in a Norwegian genome-wide study are replicated in a large European cohort.. J Psychiatr Res.

[13] Ferreira MA, O'Donovan MC, Meng YA, Jones IR, Ruderfer DM (2008). Collaborative genome-wide association analysis supports a role for ANK3 and CACNA1C in bipolar disorder.. Nat Genet.

[14] Yoshii A, Constantine-Paton M (2010). Postsynaptic BDNF-TrkB signaling in synapse maturation, plasticity, and disease.. Dev Neurobiol.

[15] Ortega JA, Alcantara S (2009). BDNF/MAPK/ERK-Induced BMP7 Expression in the Developing Cerebral Cortex Induces Premature Radial Glia Differentiation and Impairs Neuronal Migration..

[16] Bouilleret V, Loup F, Kiener T, Marescaux C, Fritschy JM (2000). Early loss of interneurons and delayed subunit-specific changes in GABA(A)-receptor expression in a mouse model of mesial temporal lobe epilepsy.. Hippocampus.

[17] Pirker S, Schwarzer C, Czech T, Baumgartner C, Pockberger H (2003). Increased expression of GABA(A) receptor beta-subunits in the hippocampus of patients with temporal lobe epilepsy.. J Neuropathol Exp Neurol.

[18] Ding L, Feng HJ, Macdonald RL, Botzolakis EJ, Hu N (2010). The GABA-A receptor alpha 1 subunit mutation A322D associated with autosomal dominant juvenile myoclonic epilepsy reduces the expression and alters the composition of wild type GABA-A receptors.. J Biol Chem.

[19] Cossette P, Liu L, Brisebois K, Dong H, Lortie A (2002). Mutation of GABRA1 in an autosomal dominant form of juvenile myoclonic epilepsy.. Nat Genet.

[20] Roberts DS, Hu Y, Lund IV, Brooks-Kayal AR, Russek SJ (2006). Brain-derived neurotrophic factor (BDNF)-induced synthesis of early growth response factor 3 (Egr3) controls the levels of type A GABA receptor alpha 4 subunits in hippocampal neurons.. J Biol Chem.

[21] Fisher RS, van Emde Boas W, Blume W, Elger C, Genton P (2005). Epileptic seizures and epilepsy: definitions proposed by the International League Against Epilepsy (ILAE) and the International Bureau for Epilepsy (IBE).. Epilepsia.

[22] Fucic A, Miskov S, Zeljezic D, Bogdanovic N, Katic J (2009). Is the role of estrogens and estrogen receptors in epilepsy still underestimated?. Med Hypotheses.

[23] Ikeda T, Matsuki N, Yamada MK (2006). Estrogen produced in cultured hippocampal neurons is a functional regulator of a GABAergic machinery.. J Neurosci Res.

[24] Jacobsen NW, Halling-Sorensen B, Birkved FK (2008). Inhibition of human aromatase complex (CYP19) by antiepileptic drugs.. Toxicol In Vitro.

[25] Isojarvi J (2008). Disorders of reproduction in patients with epilepsy: antiepileptic drug related mechanisms.. Seizure.

[26] Winum J-Y, Scozzafava A, Montero J-L, Supuran CT (2004). Therapeutic applications of sulfamates.. Expert Opinion on Therapeutic Patents.

[27] Lerner JT, Sankar R, Mazarati AM (2008). Galanin and epilepsy.. Cell Mol Life Sci.

[28] Kimura N, Takamatsu N, Yaoita Y, Osamura RY (2008). Identification of transcriptional regulatory elements in the human somatostatin receptor sst2 promoter and regions including estrogen response element half-site for estrogen activation.. J Mol Endocrinol.

[29] Smialowska M, Domin H, Zieba B, Kozniewska E, Michalik R (2009). Neuroprotective effects of neuropeptide Y-Y2 and Y5 receptor agonists in vitro and in vivo.. Neuropeptides.

[30] Colmers WF, El Bahh B (2003). Neuropeptide Y and Epilepsy.. Epilepsy Curr.

[31] Allen NC, Bagade S, McQueen MB, Ioannidis JP, Kavvoura FK (2008). Systematic meta-analyses and field synopsis of genetic association studies in schizophrenia: the SzGene database.. Nat Genet.

[32] Cascella NG, Schretlen DJ, Sawa A (2009). Schizophrenia and epilepsy: is there a shared susceptibility?. Neurosci Res.

[33] Stephens SH, Logel J, Barton A, Franks A, Schultz J (2009). Association of the 5′-upstream regulatory region of the alpha7 nicotinic acetylcholine receptor subunit gene (CHRNA7) with schizophrenia.. Schizophr Res.

[34] Heinzen EL, Need AC, Hayden KM, Chiba-Falek O, Roses AD (2010). Genome-wide scan of copy number variation in late-onset Alzheimer's disease.. J Alzheimers Dis.

[35] Sharp AJ, Mefford HC, Li K, Baker C, Skinner C (2008). A recurrent 15q13.3 microdeletion syndrome associated with mental retardation and seizures.. Nat Genet.

[36] Miller DT, Shen Y, Weiss LA, Korn J, Anselm I (2009). Microdeletion/duplication at 15q13.2q13.3 among individuals with features of autism and other neuropsychiatric disorders.. J Med Genet.

[37] Sun J, Jia P, Fanous AH, Webb BT, van den Oord EJ (2009). A multi-dimensional evidence-based candidate gene prioritization approach for complex diseases-schizophrenia as a case.. Bioinformatics.

[38] Jia P, Zheng S, Long J, Zheng W, Zhao Z (2011). dmGWAS: dense module searching for genome-wide association studies in protein-protein interaction networks.. Bioinformatics.

[39] Baranzini SE, Galwey NW, Wang J, Khankhanian P, Lindberg R (2009). Pathway and network-based analysis of genome-wide association studies in multiple sclerosis.. Hum Mol Genet.

[40] Aerts S, Lambrechts D, Maity S, Van Loo P, Coessens B (2006). Gene prioritization through genomic data fusion.. Nat Biotechnol.

