# Supplementary material for: Prioritization of Epilepsy Associated Candidate Genes by Convergent Analysis
Source: PLoS One. 2011 Feb 24;6(2):e17162. doi: 10.1371/journal.pone.0017162 (PMC3044734; doi:10.1371/journal.pone.0017162)
Supplement: Table S1 — Significant pathways in CNV-subnetwork. (DOC) [file pone.0017162.s001.doc]

**Table S1** Significant pathways in CNV-subnetwork

| **Ingenuity canonical pathways** | **-log(P-value)** | **P-value** | **Ratio** | **Molecules** |
| --- | --- | --- | --- | --- |
| Molecular Mechanisms of Cancer | 9.66 | 2.188×10^-10^ | 0.070 | MAPK1, PIK3R1, JAK2, MYC, RB1, NAIP, SUFU, MAP3K7, GNA13, CTNNB1, BRCA1, SMAD1, TP53, SMAD2, PRKDC, CASP3, SMAD9, CREBBP, GNAI1, MAPK8, PIK3R3, PRKCD, PRKACA, NOTCH1, CDK2, PRKCB |
| Glucocorticoid Receptor Signaling | 9.18 | 6.607×10^-10^ | 0.079 | TAF9, SMAD2, VCAM1, MAPK1, CDK7, PIK3R1, CREBBP, MAPK8, GTF2H2, JAK2, HSPA5, IL13, MNAT1, PIK3R3, HSPA8, TRAF2, MAP3K7, SMARCA2, VIPR1, PRKACA, HSP90AA1, ESR1 |
| RAR Activation | 8.27 | 5.370×10^-9^ | 0.094 | SMAD2, SMAD9, CDK7, PIK3R1, CREBBP, MAPK8, GTF2H2, JAK2, RBP3, MNAT1, PIK3R3, SMARCA2, PRKCD, PRKACA, RXRA, SMAD1, PRKCB |
| Cell Cycle: G2/M DNA Damage Checkpoint Regulation | 7.71 | 1.949×10^-8^ | 0.209 | TP53, PRKDC, UBB, CCNB1, YWHAE, YWHAB, CDK7, YWHAZ, BRCA1 |
| Myc Mediated Apoptosis Signaling | 6.99 | 1.023×10^-7^ | 0.159 | PIK3R3, TP53, MYC, YWHAG, CASP3, YWHAE, YWHAB, PIK3R1, MAPK8, YWHAZ |
| PI3K/AKT Signaling | 6.75 | 1.778×10^-7^ | 0.095 | ITGB1, TP53, CDC37, YWHAG, YWHAE, MAPK1, YWHAB, PIK3R1, YWHAZ, JAK2, PIK3R3, HSP90AA1, CTNNB1 |
| Androgen Signaling | 6.18 | 6.607×10^-7^ | 0.083 | MAPK1, PRKCD, CDK7, CREBBP, GNAI1, PRKACA, GTF2H2, HSP90AA1, NCOA4, GNA13, MNAT1, PRKCB |
| Type II Diabetes Mellitus Signaling | 6.14 | 7.244×10^-7^ | 0.076 | PIK3R3, TRAF2, PRKAB2, PRKAB1, MAPK1, MAP3K7, PRKCD, PIK3R1, MAPK8, CD36, TNFRSF1B, PRKCB |
| PPARα/RXRα Activation | 5.87 | 1.349×10^-6^ | 0.077 | SMAD2, PRKAB2, PRKAB1, MAPK1, CREBBP, MAPK8, CD36, JAK2, ABCA1, MAP3K7, PRKACA, HSP90AA1, RXRA, PRKCB |
| NF-κB Signaling | 5.76 | 1.738×10^-6^ | 0.086 | PIK3R1, CREBBP, MAPK8, PIK3R3, TLR10, TRAF2, CD40, MAP3K7, PRKACA, TLR3, TNFRSF1B, MAP3K3, PRKCB |
| Pancreatic Adenocarcinoma Signaling | 5.70 | 1.995×10^-6^ | 0.095 | RAD51, PIK3R3, TP53, SMAD2, RB1, MAPK1, PIK3R1, MAPK8, JAK2, NOTCH1, CDK2 |
| Axonal Guidance Signaling | 5.67 | 2.138×10^-6^ | 0.052 | SEMA3E, ITGB1, NTF3, MAPK1, ADAM22, BDNF, CXCR4, PIK3R1, GNAI1, PLXND1, BCAR1, PIK3R3, NCK2, SEMA3A, SUFU, PRKCD, PRKACA, SEMA3C, GNA13, NRP1, PRKCB |
| ERK/MAPK Signaling | 5.53 | 2.951×10^-6^ | 0.073 | ITGB1, YWHAG, MAPK1, YWHAB, PIK3R1, YWHAZ, BCAR1, MYC, PIK3R3, PRKCD, PRKACA, PPP1CA, ESR1, PRKCB |
| 14-3-3-mediated Signaling | 5.41 | 3.890×10^-6^ | 0.097 | PIK3R3, TRAF2, YWHAG, YWHAE, MAPK1, YWHAB, PRKCD, PIK3R1, MAPK8, YWHAZ, PRKCB |
| p53 Signaling | 5.39 | 4.074×10^-6^ | 0.109 | CCNG1, PIK3R3, TP53, PRKDC, RB1, PIK3R1, MAPK8, BRCA1, CTNNB1, CDK2 |
| Leukocyte Extravasation Signaling | 5.28 | 5.248×10^-6^ | 0.072 | ITGB1, VCAM1, CXCR4, MMP14, PIK3R1, ACTA2, MAPK8, GNAI1, BCAR1, PIK3R3, CLDN12, PRKCD, CTNNB1, PRKCB |
| Thyroid Cancer Signaling | 5.25 | 5.623×10^-6^ | 0.167 | TP53, MYC, NTF3, MAPK1, BDNF, RXRA, CTNNB1 |
| Neuropathic Pain Signaling In Dorsal Horn Neurons | 5.17 | 6.760×10^-6^ | 0.097 | PIK3R3, MAPK1, GPR37, BDNF, GRM8, GRM3, PRKCD, PIK3R1, PRKACA, PRKCB |
| HGF Signaling | 5.09 | 8.128×10^-6^ | 0.097 | PIK3R3, MAPK1, MAP3K7, PRKCD, PIK3R1, HGF, MAPK8, MAP3K3, CDK2, PRKCB |
| Acute Phase Response Signaling | 5.05 | 8.912×10^-6^ | 0.073 | MAPK1, PIK3R1, MAPK8, VWF, JAK2, RBP3, PIK3R3, KLKB1, TRAF2, SOD2, F8, MAP3K7, TNFRSF1B |
| p70S6K Signaling | 5.01 | 9.772×10^-6^ | 0.084 | PIK3R3, YWHAG, YWHAE, MAPK1, YWHAB, PRKCD, PIK3R1, YWHAZ, GNAI1, CD79A, PRKCB |
| Prostate Cancer Signaling | 4.95 | 1.222×10^-5^ | 0.094 | PIK3R3, TP53, RB1, MAPK1, PIK3R1, CREBBP, HSP90AA1, CTNNB1, CDK2 |
| Production of Nitric Oxide and Reactive Oxygen Species in Macrophages | 4.79 | 1.622×10^-5^ | 0.065 | PIK3R3, MAPK1, MAP3K7, PRKCD, PIK3R1, CREBBP, MAPK8, JAK2, TNFRSF1B, PPP1CA, MAP3K3, PRKCB |
| Hepatic Cholestasis | 4.68 | 2.089×10^-5^ | 0.066 | ABCB1, TRAF2, ABCB4, MAP3K7, PRKCD, MAPK8, PRKACA, TNFRSF1B, RXRA, ESR1, PRKCB |
| Prolactin Signaling | 4.60 | 2.512×10^-5^ | 0.107 | PIK3R3, MYC, MAPK1, PRKCD, PIK3R1, CREBBP, JAK2, PRKCB |
| Thrombopoietin Signaling | 4.53 | 2.951×10^-5^ | 0.119 | PIK3R3, MYC, MAPK1, PRKCD, PIK3R1, JAK2, PRKCB |
| Small Cell Lung Cancer Signaling | 4.46 | 3.467×10^-5^ | 0.090 | PIK3R3, TP53, MYC, RB1, TRAF2, PIK3R1, RXRA, CDK2 |
| IGF-1 Signaling | 4.45 | 3.548×10^-5^ | 0.090 | PIK3R3, YWHAG, YWHAE, MAPK1, YWHAB, PIK3R1, MAPK8, YWHAZ, PRKACA |
| Coagulation System | 4.38 | 4.169×10^-5^ | 0.162 | F11, KNG1 (includes EG:3827), KLKB1, F8, PROS1, VWF |
| Hereditary Breast Cancer Signaling | 4.37 | 4.266×10^-5^ | 0.078 | RAD51, PIK3R3, TP53, UBB, RB1, CCNB1, SMARCA2, PIK3R1, CREBBP, BRCA1 |
| Lymphotoxin β Receptor Signaling | 4.37 | 4.266×10^-5^ | 0.115 | PIK3R3, TRAF2, VCAM1, CASP3, MAPK1, PIK3R1, CREBBP |
| NRF2-mediated Oxidative Stress Response | 4.28 | 5.248×10^-5^ | 0.066 | PIK3R3, UBB, USP14, SOD2, MAPK1, MAP3K7, PRKCD, PIK3R1, ACTA2, CREBBP, MAPK8, PRKCB |
| Colorectal Cancer Metastasis Signaling | 4.22 | 6.026×10^-5^ | 0.056 | TP53, SMAD2, CASP3, MAPK1, MMP14, PIK3R1, MAPK8, JAK2, PIK3R3, MYC, TLR10, PRKACA, TLR3, CTNNB1 |
| TGF-β Signaling | 4.20 | 6.310×10^-5^ | 0.096 | SMAD2, UBB, MAPK1, SMAD9, MAP3K7, CREBBP, MAPK8, SMAD1 |
| Aldosterone Signaling in Epithelial Cells | 4.16 | 6.918×10^-5^ | 0.084 | PIK3R3, HSPA8, MAPK1, PRKCD, PIK3R1, HSP90AA1, HSPA5, PRKCB |
| Relaxin Signaling | 4.13 | 7.413×10^-5^ | 0.067 | PIK3R3, MAPK1, RLN1, PIK3R1, RXFP1, GNAI1, PRKACA, RLN2, RLN3, GNA13 |
| Induction of Apoptosis by HIV1 | 4.11 | 7.762×10^-5^ | 0.108 | TP53, TRAF2, NAIP, CASP3, CXCR4, MAPK8, TNFRSF1B |
| ILK Signaling | 4.11 | 7.762×10^-5^ | 0.065 | ITGB1, NCK2, PIK3R3, MYC, MAPK1, FLNA, PIK3R1, ACTA2, MAPK8, MYH9, MYH11, CTNNB1 |
| HIF1α Signaling | 4.10 | 7.943×10^-5^ | 0.083 | PIK3R3, TP53, MAPK1, MMP14, PIK3R1, CREBBP, MAPK8, HSP90AA1, VHL |
| Role of Osteoblasts, Osteoclasts and Chondrocytes in Rheumatoid Arthritis | 4.07 | 8.511×10^-5^ | 0.057 | ITGB1, MAPK1, SMAD9, MMP14, PIK3R1, MAPK8, PIK3R3, TRAF2, NAIP, MAP3K7, TNFRSF1B, CTNNB1, SMAD1 |
| CD40 Signaling | 4.07 | 8.511×10^-5^ | 0.104 | PIK3R3, TRAF2, CD40, MAPK1, MAP3K7, PIK3R1, MAPK8 |
| Factors Promoting Cardiogenesis in Vertebrates | 4.05 | 8.913×10^-5^ | 0.090 | SMAD2, SMAD9, MAP3K7, PRKCD, CTNNB1, SMAD1, CDK2, PRKCB |
| Aryl Hydrocarbon Receptor Signaling | 3.88 | 1.318×10^-4^ | 0.065 | TP53, MYC, RB1, NFIC, MAPK1, MAPK8, HSP90AA1, RXRA, ESR1, CDK2 |
| FAK Signaling | 3.86 | 1.380×10^-4^ | 0.082 | ITGB1, PIK3R3, MAPK1, HMMR, PIK3R1, ACTA2, BCAR1, GIT2 |
| Huntington's Disease Signaling | 3.83 | 1.479×10^-4^ | 0.054 | TP53, UBB, CASP3, MAPK1, BDNF, PIK3R1, CREBBP, MAPK8, HSPA5, PIK3R3, HSPA8, PRKCD, PRKCB |
| Neuregulin Signaling | 3.76 | 1.738×10^-4^ | 0.079 | ITGB1, PIK3R3, MYC, MAPK1, PRKCD, PIK3R1, HSP90AA1, PRKCB |
| CREB Signaling in Neurons | 3.75 | 1.778×10^-4^ | 0.056 | PIK3R3, MAPK1, GRM8, GRM3, PRKCD, PIK3R1, CREBBP, GNAI1, PRKACA, GNA13, PRKCB |
| Endothelin-1 Signaling | 3.73 | 1.862×10^-4^ | 0.060 | PIK3R3, MYC, CASP3, MAPK1, PRKCD, PIK3R1, MAPK8, YWHAZ, GNAI1, GNA13, PRKCB |
| Renal Cell Carcinoma Signaling | 3.72 | 1.905×10^-4^ | 0.097 | PIK3R3, UBB, MAPK1, PIK3R1, HGF, CREBBP, VHL |
| ERK5 Signaling | 3.72 | 1.905×10^-4^ | 0.099 | MYC, YWHAG, YWHAE, YWHAB, YWHAZ, GNA13, MAP3K3 |
| PDGF Signaling | 3.72 | 1.905×10^-4^ | 0.092 | PIK3R3, MYC, MAPK1, PIK3R1, MAPK8, JAK2, PRKCB |
| SAPK/JNK Signaling | 3.69 | 2.042×10^-4^ | 0.082 | PIK3R3, TP53, TRAF2, MAP3K7, PIK3R1, MAPK8, GNA13, MAP3K3 |
| LPS-stimulated MAPK Signaling | 3.68 | 2.089×10^-4^ | 0.090 | PIK3R3, MAPK1, MAP3K7, PRKCD, PIK3R1, MAPK8, PRKCB |
| Fcγ Receptor-mediated Phagocytosis in Macrophages and Monocytes | 3.66 | 2.188×10^-4^ | 0.079 | NCK2, PIK3R3, YES1, MAPK1, PRKCD, PIK3R1, ACTA2, PRKCB |
| Protein Kinase A Signaling | 3.61 | 2.455×10^-4^ | 0.048 | YWHAG, YWHAE, MAPK1, YWHAB, CREBBP, YWHAZ, GNAI1, AKAP3, FLNA, PRKCD, PRKACA, GNA13, PPP1CA, CTNNB1, PRKCB |
| ATM Signaling | 3.58 | 2.630×10^-4^ | 0.113 | RAD51, TP53, CCNB1, MAPK8, BRCA1, CDK2 |
| NF-κB Activation by Viruses | 3.56 | 2.754×10^-4^ | 0.086 | ITGB1, PIK3R3, TRAF2, MAPK1, PRKCD, PIK3R1, PRKCB |
| BMP signaling pathway | 3.56 | 2.754×10^-4^ | 0.088 | MAPK1, SMAD9, MAP3K7, CREBBP, MAPK8, PRKACA, SMAD1 |
| Endometrial Cancer Signaling | 3.53 | 2.951×10^-4^ | 0.105 | PIK3R3, TP53, MYC, MAPK1, PIK3R1, CTNNB1 |
| GNRH Signaling | 3.50 | 3.162×10^-4^ | 0.063 | MAPK1, MAP3K7, PRKCD, CREBBP, MAPK8, GNAI1, PRKACA, MAP3K3, PRKCB |
| Germ Cell-Sertoli Cell Junction Signaling | 3.50 | 3.162×10^-4^ | 0.063 | ITGB1, PIK3R3, MAPK1, MAP3K7, PIK3R1, ACTA2, MAPK8, CTNNB1, MAP3K3, BCAR1 |
| CXCR4 Signaling | 3.45 | 3.548×10^-4^ | 0.060 | PIK3R3, MAPK1, CXCR4, PRKCD, PIK3R1, MAPK8, GNAI1, GNA13, BCAR1, PRKCB |
| Ovarian Cancer Signaling | 3.43 | 3.715×10^-4^ | 0.067 | RAD51, PIK3R3, TP53, RB1, MAPK1, PIK3R1, PRKACA, BRCA1, CTNNB1 |
| Breast Cancer Regulation by Stathmin1 | 3.38 | 4.169×10^-4^ | 0.055 | PIK3R3, TP53, MAPK1, PRKCD, PIK3R1, GNAI1, PRKACA, GNA13, PPP1CA, CDK2, PRKCB |
| Hepatic Fibrosis / Hepatic Stellate Cell Activation | 3.35 | 4.467×10^-4^ | 0.067 | SMAD2, VCAM1, CD40, FGFR1, HGF, ACTA2, MYH9, MYH11, TNFRSF1B |
| Synaptic Long Term Potentiation | 3.32 | 4.786×10^-4^ | 0.071 | MAPK1, GRM8, GRM3, PRKCD, CREBBP, PRKACA, PPP1CA, PRKCB |
| Renin-Angiotensin Signaling | 3.32 | 4.786×10^-4^ | 0.067 | PIK3R3, MAPK1, PRKCD, PIK3R1, MAPK8, PRKACA, JAK2, PRKCB |
| TREM1 Signaling | 3.31 | 4.898×10^-4^ | 0.087 | ITGB1, TLR10, CD40, MAPK1, JAK2, TLR3 |
| FGF Signaling | 3.22 | 6.026×10^-4^ | 0.080 | PIK3R3, MAPK1, PIK3R1, FGFR1, HGF, MAPK8, FGF6 |
| Role of PKR in Interferon Induction and Antiviral Response | 3.18 | 6.607×10^-4^ | 0.109 | TP53, TRAF2, CASP3, MAP3K7, TLR3 |
| Role of NANOG in Mammalian Embryonic Stem Cell Pluripotency | 3.16 | 6.918×10^-4^ | 0.070 | PIK3R3, TP53, MAPK1, SMAD9, PIK3R1, JAK2, CTNNB1, SMAD1 |
| Estrogen Receptor Signaling | 3.13 | 7.413×10^-4^ | 0.067 | TAF9, PRKDC, MAPK1, CDK7, CREBBP, GTF2H2, MNAT1, ESR1 |
| IL-12 Signaling and Production in Macrophages | 3.11 | 7.762×10^-4^ | 0.060 | PIK3R3, CD40, MAPK1, PRKCD, PIK3R1, MAPK8, RXRA, PRKCB |
| Virus Entry via Endocytic Pathways | 3.09 | 8.128×10^-4^ | 0.073 | ITGB1, PIK3R3, FLNA, PRKCD, PIK3R1, ACTA2, PRKCB |
| PAK Signaling | 3.09 | 8.128×10^-4^ | 0.069 | ITGB1, NCK2, PIK3R3, CASP3, MAPK1, PIK3R1, MAPK8 |
| Melanoma Signaling | 3.08 | 8.318×10^-4^ | 0.109 | PIK3R3, TP53, RB1, MAPK1, PIK3R1 |
| Growth Hormone Signaling | 3.08 | 8.318×10^-4^ | 0.086 | PIK3R3, MAPK1, PRKCD, PIK3R1, JAK2, PRKCB |
| PPAR Signaling | 3.06 | 8.710×10^-4^ | 0.071 | TRAF2, MAPK1, MAP3K7, CREBBP, HSP90AA1, TNFRSF1B, RXRA |
| IL-6 Signaling | 3.06 | 8.710×10^-4^ | 0.075 | ABCB1, TRAF2, MAPK1, MAP3K7, MAPK8, JAK2, TNFRSF1B |
| Macropinocytosis Signaling | 3.04 | 9.120×10^-4^ | 0.083 | ITGB1, PIK3R3, PRKCD, PIK3R1, HGF, PRKCB |
| RANK Signaling in Osteoclasts | 3.03 | 9.333×10^-4^ | 0.073 | PIK3R3, TRAF2, MAPK1, MAP3K7, PIK3R1, MAPK8, MAP3K3 |
| Erythropoietin Signaling | 3.00 | 1.000×10^-3^ | 0.079 | PIK3R3, MAPK1, PRKCD, PIK3R1, JAK2, PRKCB |
| Non-Small Cell Lung Cancer Signaling | 3.00 | 1.000×10^-3^ | 0.076 | PIK3R3, TP53, RB1, MAPK1, PIK3R1, RXRA |
| PTEN Signaling | 3.00 | 1.000×10^-3^ | 0.068 | ITGB1, PIK3R3, CASP3, MAPK1, PIK3R1, MAGI2, BCAR1 |
| Glioblastoma Multiforme Signaling | 2.96 | 1.096×10^-3^ | 0.055 | PIK3R3, TP53, MYC, RB1, MAPK1, PRKCD, PIK3R1, CTNNB1, CDK2 |
| Neurotrophin/TRK Signaling | 2.93 | 1.175×10^-3^ | 0.080 | PIK3R3, NTF3, MAPK1, BDNF, PIK3R1, MAPK8 |
| Actin Cytoskeleton Signaling | 2.89 | 1.288×10^-3^ | 0.047 | ITGB1, PIK3R3, MAPK1, PIK3R1, ACTA2, MYH9, MYH11, GNA13, PPP1CA, BCAR1, FGF6 |
| LXR/RXR Activation | 2.87 | 1.349×10^-3^ | 0.070 | CD36, ACACA, TLR3, TNFRSF1B, RXRA, ABCA1 |
| IL-3 Signaling | 2.87 | 1.349×10^-3^ | 0.083 | PIK3R3, MAPK1, PRKCD, PIK3R1, JAK2, PRKCB |
| Melatonin Signaling | 2.87 | 1.349×10^-3^ | 0.078 | MTNR1A, MAPK1, PRKCD, GNAI1, PRKACA, PRKCB |
| Xenobiotic Metabolism Signaling | 2.85 | 1.413×10^-3^ | 0.045 | ABCB1, MAPK1, PIK3R1, CREBBP, MAPK8, GRIP1, PIK3R3, MAP3K7, PRKCD, HSP90AA1, RXRA, MAP3K3, PRKCB |
| Fc Epsilon RI Signaling | 2.84 | 1.445×10^-3^ | 0.068 | PIK3R3, MAPK1, PRKCD, PIK3R1, MAPK8, IL13, PRKCB |
| Glioma Signaling | 2.84 | 1.445×10^-3^ | 0.063 | PIK3R3, TP53, RB1, MAPK1, PRKCD, PIK3R1, PRKCB |
| Chemokine Signaling | 2.80 | 1.585×10^-3^ | 0.080 | MAPK1, CXCR4, MAPK8, GNAI1, PPP1CA, PRKCB |
| IL-17 Signaling | 2.77 | 1.698×10^-3^ | 0.081 | PIK3R3, MAPK1, MAP3K7, PIK3R1, MAPK8, JAK2 |
| Role of Macrophages, Fibroblasts and Endothelial Cells in Rheumatoid Arthritis | 2.76 | 1.738×10^-3^ | 0.041 | VCAM1, MAPK1, PIK3R1, JAK2, PIK3R3, MYC, TLR10, TRAF2, MAP3K7, PRKCD, TLR3, TNFRSF1B, CTNNB1, PRKCB |
| Human Embryonic Stem Cell Pluripotency | 2.75 | 1.778×10^-3^ | 0.054 | PIK3R3, SMAD2, NTF3, BDNF, PIK3R1, FGFR1, CTNNB1, SMAD1 |
| Leptin Signaling in Obesity | 2.74 | 1.820×10^-3^ | 0.073 | NPY, PIK3R3, MAPK1, PIK3R1, PRKACA, JAK2 |
| CD27 Signaling in Lymphocytes | 2.70 | 1.995×10^-3^ | 0.088 | TRAF2, CASP3, MAP3K7, MAPK8, MAP3K3 |
| Tight Junction Signaling | 2.68 | 2.089×10^-3^ | 0.054 | CLDN12, ACTA2, PRKACA, MYH9, MYH11, TNFRSF1B, MAGI2, CTNNB1, OCLN |
| Dendritic Cell Maturation | 2.68 | 2.089×10^-3^ | 0.052 | PIK3R3, CD40, MAPK1, PIK3R1, MAPK8, JAK2, TLR3, TNFRSF1B, DDR1 |
| Reelin Signaling in Neurons | 2.68 | 2.089×10^-3^ | 0.077 | ITGB1, PIK3R3, YES1, PIK3R1, MAPK8, VLDLR |
| HER-2 Signaling in Breast Cancer | 2.68 | 2.089×10^-3^ | 0.076 | ITGB1, PIK3R3, TP53, PRKCD, PIK3R1, PRKCB |
| Nitric Oxide Signaling in the Cardiovascular System | 2.65 | 2.239×10^-3^ | 0.062 | PIK3R3, KNG1 (includes EG:3827), PRKCD, PIK3R1, PRKACA, HSP90AA1 |
| Type I Diabetes Mellitus Signaling | 2.61 | 2.455×10^-3^ | 0.061 | CD28, TRAF2, CASP3, MAP3K7, MAPK8, JAK2, TNFRSF1B |
| Glioma Invasiveness Signaling | 2.59 | 2.570×10^-3^ | 0.088 | PIK3R3, MAPK1, HMMR, PIK3R1, VTN |
| IL-8 Signaling | 2.56 | 2.754×10^-3^ | 0.048 | PIK3R3, VCAM1, MAPK1, PRKCD, PIK3R1, MAPK8, GNAI1, GNA13, PRKCB |
| mTOR Signaling | 2.52 | 3.020×10^-3^ | 0.051 | PIK3R3, DGKZ, PRKAB2, PRKAB1, MAPK1, PRKCD, PIK3R1, PRKCB |
| Role of BRCA1 in DNA Damage Response | 2.52 | 3.020×10^-3^ | 0.082 | RAD51, TP53, RB1, SMARCA2, BRCA1 |
| CCR3 Signaling in Eosinophils | 2.52 | 3.020×10^-3^ | 0.058 | PIK3R3, MAPK1, PRKCD, PIK3R1, GNAI1, PPP1CA, PRKCB |
| Synaptic Long Term Depression | 2.47 | 3.388×10^-3^ | 0.050 | MAPK1, GRM8, GRM3, PRKCD, YWHAZ, GNAI1, GNA13, PRKCB |
| Death Receptor Signaling | 2.45 | 3.548×10^-3^ | 0.078 | TRAF2, NAIP, CASP3, MAPK8, TNFRSF1B |
| Apoptosis Signaling | 2.43 | 3.715×10^-3^ | 0.067 | TP53, NAIP, CASP3, MAPK1, MAPK8, TNFRSF1B |
| B Cell Receptor Signaling | 2.43 | 3.715×10^-3^ | 0.052 | PIK3R3, MAPK1, MAP3K7, PIK3R1, MAPK8, MAP3K3, CD79A, PRKCB |
| VEGF Signaling | 2.41 | 3.890×10^-3^ | 0.062 | PIK3R3, YWHAE, MAPK1, PIK3R1, ACTA2, PRKCB |
| TR/RXR Activation | 2.38 | 4.169×10^-3^ | 0.062 | PIK3R3, PIK3R1, NCOA4, ACACA, GRIP1, RXRA |
| Bladder Cancer Signaling | 2.36 | 4.365×10^-3^ | 0.067 | TP53, MYC, RB1, MAPK1, MMP14, FGF6 |
| IL-15 Signaling | 2.36 | 4.365×10^-3^ | 0.075 | PIK3R3, TRAF2, MAPK1, PIK3R1, JAK2 |
| GM-CSF Signaling | 2.33 | 4.677×10^-3^ | 0.075 | PIK3R3, MAPK1, PIK3R1, JAK2, PRKCB |
| Role of NFAT in Cardiac Hypertrophy | 2.32 | 4.786×10^-3^ | 0.044 | PIK3R3, MAPK1, MAP3K7, PRKCD, PIK3R1, MAPK8, GNAI1, PRKACA, PRKCB |
| G Beta Gamma Signaling | 2.26 | 5.495×10^-3^ | 0.050 | MAPK1, PRKCD, GNAI1, PRKACA, GNA13, PRKCB |
| HMGB1 Signaling | 2.24 | 5.754×10^-3^ | 0.061 | PIK3R3, VCAM1, MAPK1, PIK3R1, MAPK8, TNFRSF1B |
| Cardiac Hypertrophy Signaling | 2.22 | 6.026×10^-3^ | 0.041 | PIK3R3, MAPK1, MAP3K7, PIK3R1, CREBBP, MAPK8, GNAI1, PRKACA, GNA13, MAP3K3 |
| Chronic Myeloid Leukemia Signaling | 2.19 | 6.457×10^-3^ | 0.057 | PIK3R3, TP53, MYC, RB1, MAPK1, PIK3R1 |
| Integrin Signaling | 2.16 | 6.918×10^-3^ | 0.045 | ITGB1, NCK2, PIK3R3, MAPK1, PIK3R1, ACTA2, MAPK8, PPP1CA, BCAR1 |
| Cholecystokinin/Gastrin-mediated Signaling | 2.09 | 8.128×10^-3^ | 0.058 | MAPK1, PRKCD, MAPK8, GNA13, BCAR1, PRKCB |
| Insulin Receptor Signaling | 2.08 | 8.318×10^-3^ | 0.050 | PIK3R3, MAPK1, PIK3R1, MAPK8, PRKACA, JAK2, PPP1CA |
| AMPK Signaling | 2.06 | 8.710×10^-3^ | 0.042 | PIK3R3, PRKAB2, PRKAB1, SMARCA2, PIK3R1, PRKACA, ACACA |
| Sphingosine-1-phosphate Signaling | 2.05 | 8.913×10^-3^ | 0.054 | PIK3R3, CASP3, MAPK1, PIK3R1, GNAI1, GNA13 |
