# Supplementary material for: Prioritization of Epilepsy Associated Candidate Genes by Convergent Analysis
Source: PLoS One. 2011 Feb 24;6(2):e17162. doi: 10.1371/journal.pone.0017162 (PMC3044734; doi:10.1371/journal.pone.0017162)
Supplement: Table S2 — Significant pathways in HuGE-subnetwork. (DOC) [file pone.0017162.s002.doc]

**Table S2** Significant pathways in HuGE-subnetwork

| **Ingenuity canonical pathways** | **-log(P-value)** | **P-value** | **Ratio** | **Molecules** |
| --- | --- | --- | --- | --- |
| GABA Receptor Signaling | 13.60 | 2.512×10^-14^ | 0.236 | UBB, ABAT, UBQLN1, GABBR1, GPHN, GABBR2, GABRR2, GABRG2, GABRB3, GABRB1, GABRD, GABRA1, ALDH5A1 |
| Dendritic Cell Maturation | 11.30 | 5.012×10^-12^ | 0.103 | FCGR3B, IL1A, MAPK1, IL10, FCGR2A, HLA-DRB1, IL6, IGHG1, HLA-DQB1, HLA-A, IL1RN, HLA-B, IL1B, STAT1, TNF, TRA@, FCGR3A, HLA-C |
| Hepatic Cholestasis | 10.60 | 2.512×10^-11^ | 0.096 | IL8, ABCB1, IL1A, IL6, IL1R1, TAP1, ABCC2, ABCB4, IL1RN, NR1I2, PRKACA, IL1B, PRKCE, RXRA, TNF, PRKCA |
| Systemic Lupus Erythematosus Signaling | 9.16 | 6.918×10^-10^ | 0.099 | FCGR3B, IL1A, MAPK1, IL10, FCGR2A, IGHG1, IL6, HLA-A, IL1RN, HLA-B, IL1B, TNF, TRA@, FCGR3A, HLA-C |
| Communication between Innate and Adaptive Immune Cells | 9.07 | 8.511×10^-10^ | 0.135 | IL8, IL1A, IL10, IL1RN, CD4, HLA-DRB1, IL1B, IGHG1, IL6, TNF, TRA@, IL4 |
| IL-6 Signaling | 8.70 | 1.995×10^-9^ | 0.129 | IL8, ABCB1, IL1A, MAPK1, IL1RN, CSNK2A1, IL1B, CSNK2B, IL1R1, IL6, A2M, TNF |
| Graft-versus-Host Disease Signaling | 8.41 | 3.890×10^-9^ | 0.200 | IL1A, HLA-A, IL1RN, HLA-B, IL1B, IL6, TNF, TRA@, HLA-C |
| Allograft Rejection Signaling | 7.10 | 7.943×10^-8^ | 0.178 | HLA-A, IL10, HLA-B, IGHG1, TNF, TRA@, IL4, HLA-C |
| Glucocorticoid Receptor Signaling | 6.50 | 3.162×10^-7^ | 0.057 | IL8, MAPK1, IL10, SMAD3, POMC, IL6, CD163, AR, IL1RN, PRKACA, IL1B, STAT1, TNF, A2M, TRA@, IL4 |
| LXR/RXR Activation | 6.44 | 3.631×10^-7^ | 0.105 | APOE, IL1A, IL1RN, IL1B, IL1R1, IL6, RXRA, TNF, ABCA1 |
| PXR/RXR Activation | 6.33 | 4.677×10^-7^ | 0.099 | GSTM1, ABCB1, ABCC2, GSTM2, NR1I2, PRKACA, IL6, RXRA, TNF |
| Role of Cytokines in Mediating Communication between Immune Cells | 6.03 | 9.333×10^-7^ | 0.140 | IL8, IL1A, IL10, IL1RN, IL1B, IL6, TNF, IL4 |
| Hepatic Fibrosis / Hepatic Stellate Cell Activation | 5.88 | 1.318×10^-6^ | 0.082 | IL8, IL1A, IL10, SMAD3, IL1B, IL1R1, IL6, STAT1, A2M, TNF, IL4 |
| IL-10 Signaling | 5.86 | 1.380×10^-6^ | 0.114 | IL1A, IL10, FCGR2A, IL1RN, IL1B, IL1R1, IL6, TNF |
| Autoimmune Thyroid Disease Signaling | 5.72 | 1.905×10^-6^ | 0.149 | HLA-A, IL10, HLA-B, IGHG1, TRA@, IL4, HLA-C |
| LPS/IL-1 Mediated Inhibition of RXR Function | 5.59 | 2.570×10^-6^ | 0.061 | GSTM1, ABCB1, APOE, GSTM3 (includes EG:2947), IL1R1, ABCA1, ABCC2, GSTM2, NR1I2, IL1B, RXRA, TNF, ALDH5A1 |
| FXR/RXR Activation | 5.53 | 2.951×10^-6^ | 0.087 | APOE, ABCC2, IL1A, ABCB4, IL1RN, NR1I2, IL1B, RXRA, TNF |
| G Beta Gamma Signaling | 5.41 | 3.890×10^-6^ | 0.076 | GNAI2, MAPK1, GNA15, KCNJ9, PRKACA, PRKCE, KCNJ3, KCNJ6, PRKCA |
| Xenobiotic Metabolism Signaling | 5.21 | 6.166×10^-6^ | 0.051 | GSTM1, ABCB1, IL1A, MAPK1, GSTM3 (includes EG:2947), IL6, ABCC2, GSTM2, NR1I2, IL1B, PRKCE, RXRA, TNF, ALDH5A1, PRKCA |
| Amyloid Processing | 5.11 | 7.762×10^-6^ | 0.119 | MAPK1, CSNK2A1, PRKACA, PRKCE, BACE1, CSNK2B, APP |
| Antigen Presentation Pathway | 5.10 | 7.943×10^-6^ | 0.154 | HLA-A, HLA-B, HLA-DRB1, CANX, TAP1, HLA-C |
| TREM1 Signaling | 4.95 | 1.222×10^-5^ | 0.101 | IL8, MAPK1, IL10, CASP1, IL1B, IL6, TNF |
| Aryl Hydrocarbon Receptor Signaling | 4.85 | 1.413×10^-5^ | 0.065 | GSTM1, IL1A, GSTM2, MAPK1, GSTM3 (includes EG:2947), IL1B, IL6, RXRA, TNF, ALDH5A1 |
| Altered T Cell and B Cell Signaling in Rheumatoid Arthritis | 4.83 | 1.479×10^-5^ | 0.092 | IL1A, IL10, IL1RN, IL1B, IL6, TNF, TRA@, IL4 |
| Neuroprotective Role of THOP1 in Alzheimer's Disease | 4.82 | 1.514×10^-5^ | 0.111 | HLA-A, HLA-B, PRKACA, APP, ACE, HLA-C |
| Acute Phase Response Signaling | 4.74 | 1.820×10^-5^ | 0.062 | IL1A, HP, MAPK1, IL1RN, IL1B, IL1R1, IL6, A2M, TNF, HMOX2, F2 |
| Corticotropin Releasing Hormone Signaling | 4.70 | 1.995×10^-5^ | 0.066 | GNAI2, GLI3, MAPK1, BDNF, CNR1, PRKACA, PRKCE, POMC, PRKCA |
| Renin-Angiotensin Signaling | 4.11 | 7.762×10^-5^ | 0.067 | MAPK1, PRKACA, PRKCE, STAT1, TNF, AGTR2, ACE, PRKCA |
| Type I Diabetes Mellitus Signaling | 4.08 | 8.318×10^-5^ | 0.070 | HLA-A, HLA-B, IL1B, IL1R1, STAT1, TNF, TRA@, HLA-C |
| G-Protein Coupled Receptor Signaling | 3.99 | 1.023×10^-4^ | 0.050 | GNAI2, FYN, MAPK1, OPRM1, DUSP6, PRKACA, PRKCE, DRD4, DRD2, AGTR2, PRKCA |
| Role of NFAT in Regulation of the Immune Response | 3.97 | 1.072×10^-4^ | 0.051 | GNAI2, FCGR3B, FYN, MAPK1, GNA15, CSNK1G2, FCGR2A, CD4, FCGR3A, TRA@ |
| Role of Macrophages, Fibroblasts and Endothelial Cells in Rheumatoid Arthritis | 3.90 | 1.259×10^-4^ | 0.041 | IL8, IL1A, MAPK1, IL10, IL1R1, IGHG1, IL6, HP, IL1RN, IL1B, PRKCE, TNF, FCGR3A, PRKCA |
| IL-12 Signaling and Production in Macrophages | 3.88 | 1.318×10^-4^ | 0.060 | MAPK1, IL10, PRKCE, STAT1, RXRA, TNF, IL4, PRKCA |
| Androgen Signaling | 3.85 | 1.413×10^-4^ | 0.056 | GNAI2, AR, MAPK1, GNA15, SMAD3, PRKACA, PRKCE, PRKCA |
| NF-κB Signaling | 3.80 | 1.585×10^-4^ | 0.059 | IL1A, IL1RN, CSNK2A1, PRKACA, IL1B, CSNK2B, IL1R1, TNF, TRA@ |
| Virus Entry via Endocytic Pathways | 3.78 | 1.660×10^-4^ | 0.073 | FYN, HLA-A, ITSN1, HLA-B, PRKCE, PRKCA, HLA-C |
| PPAR Signaling | 3.75 | 1.778×10^-4^ | 0.071 | IL1A, MAPK1, IL1RN, IL1B, IL1R1, RXRA, TNF |
| Huntington's Disease Signaling | 3.68 | 2.089×10^-4^ | 0.046 | HDAC6, UBB, GRIN2B, MAPK1, BDNF, CASP1, PRKCE, DLG4, STX1A, SNAP25, PRKCA |
| Fcγ Receptor-mediated Phagocytosis in Macrophages and Monocytes | 3.60 | 2.512×10^-4^ | 0.069 | FYN, MAPK1, FCGR2A, PRKCE, CRK, FCGR3A, PRKCA |
| Crosstalk between Dendritic Cells and Natural Killer Cells | 3.60 | 2.512×10^-4^ | 0.071 | HLA-A, HLA-B, HLA-DRB1, IL6, TNF, IL4, HLA-C |
| T Helper Cell Differentiation | 3.58 | 2.630×10^-4^ | 0.088 | IL10, IL6, STAT1, TNF, TRA@, IL4 |
| EGF Signaling | 3.56 | 2.754×10^-4^ | 0.102 | MAPK1, CSNK2A1, CSNK2B, STAT1, PRKCA |
| Neuropathic Pain Signaling In Dorsal Horn Neurons | 3.52 | 3.020×10^-4^ | 0.068 | GRIN2B, KCNQ2, MAPK1, BDNF, PRKACA, PRKCE, PRKCA |
| PDGF Signaling | 3.50 | 3.162×10^-4^ | 0.079 | MAPK1, CSNK2A1, CRK, CSNK2B, STAT1, PRKCA |
| Insulin Receptor Signaling | 3.41 | 3.890×10^-4^ | 0.057 | FYN, TSC1, MAPK1, TSC2, HLA-B, PRKACA, CRK, VAMP2 |
| Cholecystokinin/Gastrin-mediated Signaling | 3.41 | 3.890×10^-4^ | 0.067 | IL1A, MAPK1, IL1RN, PRKCE, IL1B, TNF, PRKCA |
| Role of Pattern Recognition Receptors in Recognition of Bacteria and Viruses | 3.40 | 3.981×10^-4^ | 0.075 | MAPK1, IL10, CASP1, IL1B, IL6, TNF |
| PPARα/RXRα Activation | 3.38 | 4.619×10^-4^ | 0.050 | MAPK1, SMAD3, PRKACA, IL1B, IL1R1, IL6, RXRA, ABCA1, PRKCA |
| Caveolar-mediated Endocytosis Signaling | 3.30 | 5.012×10^-4^ | 0.072 | FYN, HLA-A, ITSN1, HLA-B, PRKCA, HLA-C |
| Reelin Signaling in Neurons | 3.27 | 5.370×10^-4^ | 0.077 | FYN, APOE, CNR1, PAFAH1B1, APP, DCX |
| NRF2-mediated Oxidative Stress Response | 3.27 | 5.370×10^-4^ | 0.049 | GSTM1, UBB, GSTM2, MAPK1, GSTM3 (includes EG:2947), PRKCE, HERPUD1, PRKCA, EPHX1 |
| α-Adrenergic Signaling | 2.91 | 1.230×10^-3^ | 0.057 | GNAI2, MAPK1, HLA-B, PRKACA, PRKCE, PRKCA |
| cAMP-mediated Signaling | 2.90 | 1.259×10^-3^ | 0.050 | GNAI2, MAPK1, OPRM1, DUSP6, PRKACA, DRD4, DRD2, AGTR2 |
| Growth Hormone Signaling | 2.79 | 1.622×10^-3^ | 0.071 | MAPK1, PRKCE, STAT1, A2M, PRKCA |
| Fc Epsilon RI Signaling | 2.74 | 1.820×10^-3^ | 0.058 | FYN, MAPK1, PRKCE, TNF, IL4, PRKCA |
| p38 MAPK Signaling | 2.69 | 2.042×10^-3^ | 0.062 | IL1A, IL1RN, IL1B, IL1R1, STAT1, TNF |
| Role of Osteoblasts, Osteoclasts and Chondrocytes in Rheumatoid Arthritis | 2.67 | 2.138×10^-3^ | 0.040 | IL1A, MAPK1, IL10, IL1RN, IL1B, IL1R1, IL6, TNF, IL4 |
| Atherosclerosis Signaling | 2.64 | 2.291×10^-3^ | 0.054 | IL8, IL1A, IL1RN, IL1B, IL6, TNF |
| CCR5 Signaling in Macrophages | 2.64 | 2.291×10^-3^ | 0.054 | GNAI2, CD4, PRKCE, TRA@, PRKCA |
| Prolactin Signaling | 2.64 | 2.291×10^-3^ | 0.067 | FYN, MAPK1, PRKCE, STAT1, PRKCA |
| Calcium Signaling | 2.62 | 2.399×10^-3^ | 0.039 | HDAC6, GRIN2B, CHRNA4, MAPK1, CHRNB2, PRKACA, CHRNA7, CHRNA2 |
| Melatonin Signaling | 2.62 | 2.399×10^-3^ | 0.065 | GNAI2, MAPK1, PRKACA, PRKCE, PRKCA |
| ERK/MAPK Signaling | 2.56 | 2.754×10^-3^ | 0.042 | FYN, MAPK1, DUSP6, PRKACA, PRKCE, CRK, STAT1, PRKCA |
| Natural Killer Cell Signaling | 2.52 | 3.020×10^-3^ | 0.054 | FCGR3B, FYN, MAPK1, PRKCE, FCGR3A, PRKCA |
| 14-3-3-mediated Signaling | 2.42 | 3.802×10^-3^ | 0.053 | TSC1, MAPK1, TSC2, PRKCE, TNF, PRKCA |
| CXCR4 Signaling | 2.34 | 4.571×10^-3^ | 0.042 | GNAI2, MAPK1, GNA15, CD4, PRKCE, CRK, PRKCA |
| Molecular Mechanisms of Cancer | 2.24 | 5.754×10^-3^ | 0.030 | GNAI2, FYN, NF1, MAPK1, GNA15, SMAD3, PRKACA, PRKCE, CRK, RALBP1, PRKCA |
| p70S6K Signaling | 2.21 | 6.166×10^-3^ | 0.046 | GNAI2, MAPK1, PRKCE, F2, IL4, PRKCA |
| CDK5 Signaling | 2.18 | 6.607×10^-3^ | 0.054 | LAMA5, MAPK1, BDNF, PRKACA, CACNA1A |
| Neuregulin Signaling | 2.14 | 7.244×10^-3^ | 0.050 | MAPK1, PRKCE, DLG4, CRK, PRKCA |
| RAR Activation | 2.14 | 7.244×10^-3^ | 0.039 | SMAD3, CSNK2A1, PRKACA, PRKCE, CSNK2B, RXRA, PRKCA |
| IL-8 Signaling | 2.12 | 7.586×10^-3^ | 0.038 | GNAI2, IL8, MAPK1, IL8RB, PRKCE, CSTB, PRKCA |
| IL-1 Signaling | 2.10 | 7.943×10^-3^ | 0.047 | GNAI2, IL1A, GNA15, PRKACA, IL1R1 |
| HMGB1 Signaling | 2.08 | 8.318×10^-3^ | 0.051 | IL8, IL1A, MAPK1, IL1R1, TNF |
| Ephrin Receptor Signaling | 2.06 | 8.710×10^-3^ | 0.036 | GNAI2, FYN, GRIN2B, ITSN1, MAPK1, GNA15, CRK |
